# Calcified intrarenal aneurysm simulating urinary lithiasis

**DOI:** 10.31744/einstein_journal/2022RC6484

**Published:** 2022-03-22

**Authors:** Mário Henrique Elias de Mattos, Arthur Cardoso Del Papa, Antonio Corrêa Lopes

**Affiliations:** 1 Centro Universitário FMABC Santo André SP Brazil Centro Universitário FMABC, Santo André, SP, Brazil.

**Keywords:** Aneurysm, Lithiasis, Renal artery, Nephrolithiasis, Vascular calcification

## Abstract

We describe the case of a female patient with calcification in renal topography, initially diagnosed as lithiasis in the left kidney, and later attributed to calcification of intrarenal vascular aneurysm. Next, we discuss the relevance of considering such an entity in the differential diagnoses of intrarenal calcifications before choosing any form of specific interventional treatment.

## INTRODUCTION

Urinary lithiasis is a prevalent and recurrent disease, whose diagnosis is usually easily made by means of radiological investigation. The definition of which modality should be used is made by analyzing the characteristics of the calculus, patient, and the known results of the various intervention methods.^(1)^A wrong diagnosis can compromise treatment and even lead to complications. This report illustrates this situation, in which the wrong diagnosis could have evolved catastrophically, and reinforces the alert to the need for attention to the quality of the exam, and analysis of the report. In case of doubt, ratify with exams that are more accurate.

## CASE REPORT

A 71-year-old woman was referred for urological counseling due to the finding of urinary calculus in a routine ultrasound examination. Patient was asymptomatic and with no past history of urinary lithiasis. Her hypertension was controlled with regular use of candesartan cilexetil and hydrochlorothiazide.

**Figure 1:**
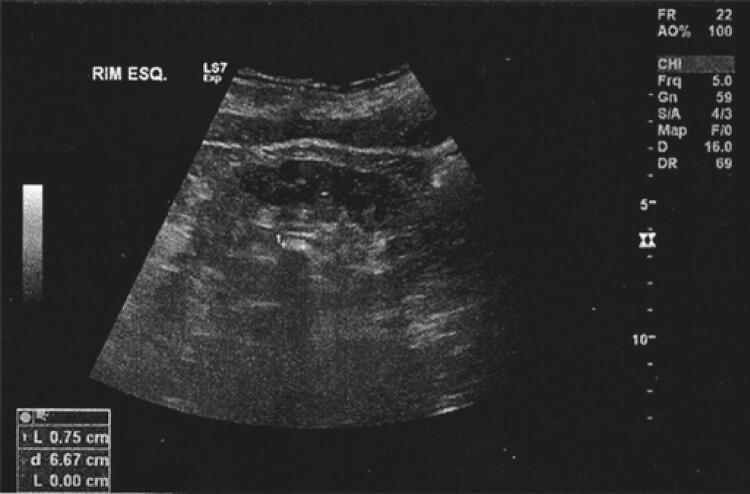
Ultrasonography suggestive of renal calculus of approximately 7.5mm in the middle third of the left kidney

She presented with recent and normal laboratory tests (blood count, urea, creatinine, urinary sediment analysis, urine culture, and metabolic study). Ultrasonography of the urinary tract showed topical kidneys with normal dimensions and a 7.5mm calculus in the middle third of the left kidney, with no dilatation of the urinary tract (Figure 1). Right kidney measured 101mm and left kidney 108mm. The bladder was unchanged.

Despite being asymptomatic, she requested that some form of treatment be considered because of her concern about developing symptoms due to calculus migration and the need for emergency treatment.

**Figure 2:**
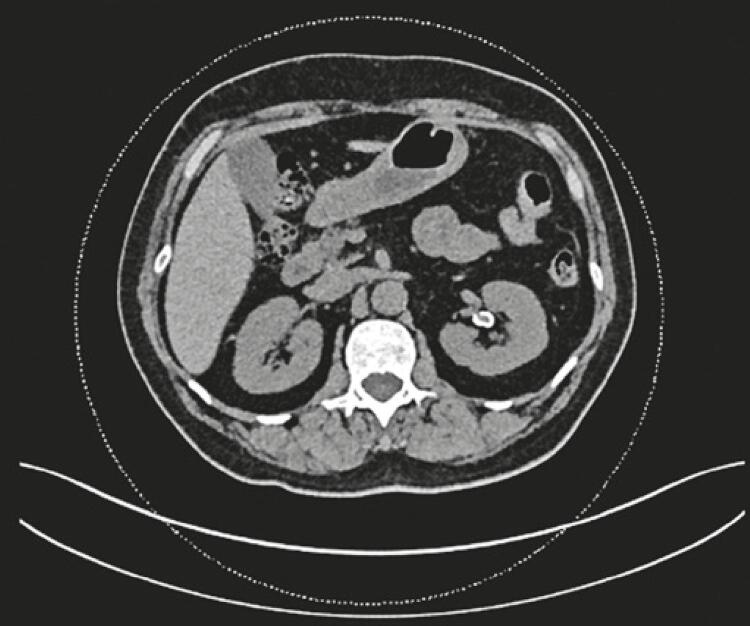
Computed tomography scan showing a saccular aneurysm with parietal calcification of the left renal artery on the plane of the renal hilum, measuring 1.0cm x 1.0cm (axial plane)

**Figure 3:**
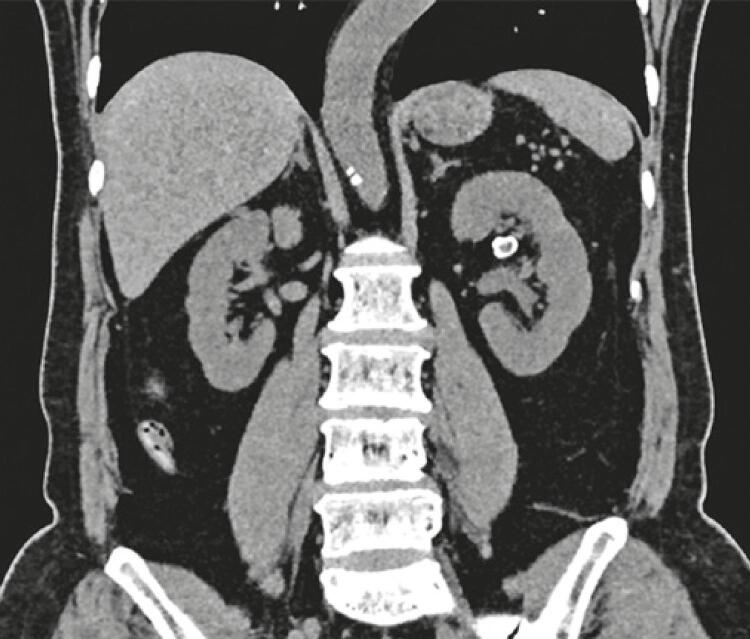
Computed tomography scan showing a saccular aneurysm with parietal calcification of the left renal artery on the plane of the renal hilum, measuring 1.0cm x 1.0cm (coronal plane)

As part of the treatment planning, a computed tomography scan was performed (without contrast), which showed an unchanged right kidney. The left kidney was unaltered, but the images suggested a saccular aneurysm with parietal calcification of the left renal artery in the plane of the renal hilum, measuring 10mm x 10mm (Figures 2 and 3). Ureters and bladder were normal.

**Figure 4:**
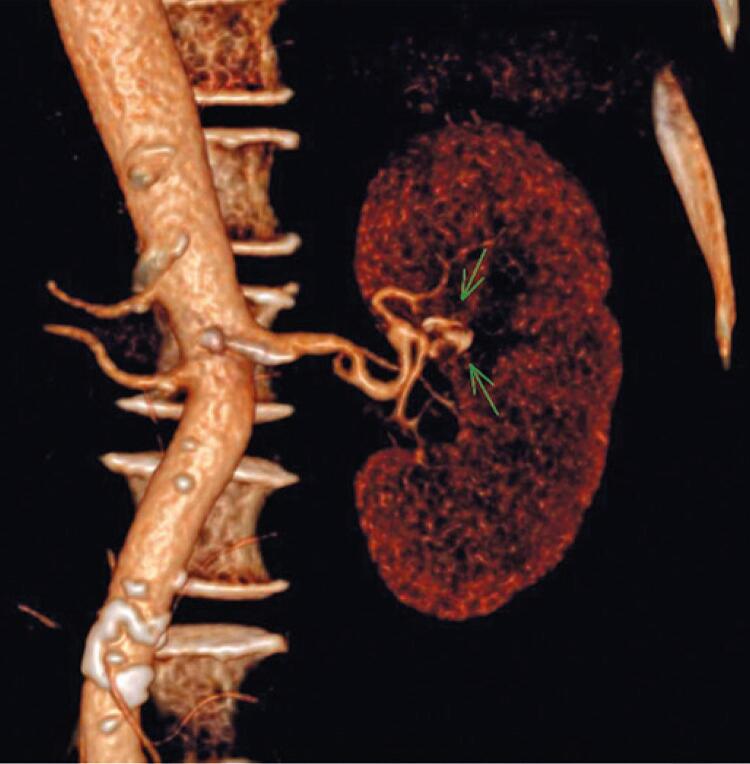
An aneurysm involving the anterior segmental branch of the left renal artery, showing a virtually complete thrombosis (three-dimensional reconstruction)

Faced with the unexpected finding, and diagnosis of urinary calculus ruled out, she was referred to a vascular surgeon. Renal artery computed tomography angiography showed that the right kidney was supplied by two arterial branches of similar caliber, patent throughout, without stenosis or aneurysm. The left kidney was supplied by a single arterial branch, with a prominent aneurysm involving the anterior segment branch, showing almost complete thrombosis, measuring 12mm x 9mm in diameter (Figure 4). The diameter of the renal artery adjacent to the aneurysm was 5mm.

The patient remains guided by the vascular surgeon only for routine follow-up and intensive control of blood pressure levels.

This study was approved by the Research Ethics Committee of *Faculdade de Medicina do ABC* under # 4.440.144, CAAE: 34350420.6.0000.0082.

## DISCUSSION

Renal artery aneurysm is an uncommon condition, with an incidence of 0.01% to 2.5% in the population, and an average growth rate of 0.06mm to 0.6mm per year. It predominates in females (72%) during the sixth decade of life. Symptomatology is poor and usually quite rare, consisting of abdominal flank pain and hematuria. It may course with hypertension, palpable and pulsatile renal mass, renal artery murmur, calcification, and thromboembolism. Its treatment is controversial, and in most cases it is conservative. However, endovascular interventional treatment may be indicated in case of rupture, or when it is understood that the risk of this event is high.^(1-3)^

There are some similar case reports of renal artery aneurysm in the literature. Rafailidis et al. reported the case of a 72-year-old patient with a renal artery aneurysm diagnosed as an ultrasound finding.^(4)^Sataa et al. reported a similar case, in which the aneurysm was misdiagnosed as renal lithiasis on ultrasound and radiography, leading to inadvertent treatment with one session of extracorporeal shock wave lithotripsy, without complications. In this case, the diagnosis of aneurysm was determined after computed tomography scan investigation in preparation for the second session.^(5)^ John demonstrated the case of a patient with calcification of bilateral renal artery aneurysm.^(6)^

The particularity in the reported case is the striking similarity of the calcified aneurysm with renal lithiasis on screening imaging. The fact that the calcified aneurysm was located in the intraparenchymal segment of the renal artery led to diagnostic confusion. A computed tomography scan to define the treatment determined a radical change in diagnosis and management. Extracorporeal shock wave lithotripsy, if chosen without recognition of the real diagnosis, could have been accompanied by hemorrhagic complications, which could have put the patient’s life at risk. This case offers some considerations on the radiological investigation in the preparation prior to the intervention. The suggestion is that in selected cases of higher complexity of urinary lithiasis, a more accurate imaging test of the urinary tract should be considered for a better diagnostic and therapeutic definition.^(7)^
